# Advancing Healthcare: Synergizing Biosensors and Machine Learning for Early Cancer Diagnosis

**DOI:** 10.3390/bios13090884

**Published:** 2023-09-13

**Authors:** Mahtab Kokabi, Muhammad Nabeel Tahir, Darshan Singh, Mehdi Javanmard

**Affiliations:** Department of Electrical and Computer Engineering, Rutgers the State University of New Jersey, Piscataway, NJ 08854, USA; mk1903@scarletmail.rutgers.edu (M.K.); nabeel.tahir@rutgers.edu (M.N.T.); ds1464@scarletmail.rutgers.edu (D.S.)

**Keywords:** biosensors, impedance cytometry, lab-on-a-chip, cancer detection, machine learning, microfluidic chips

## Abstract

Cancer is a fatal disease and a significant cause of millions of deaths. Traditional methods for cancer detection often have limitations in identifying the disease in its early stages, and they can be expensive and time-consuming. Since cancer typically lacks symptoms and is often only detected at advanced stages, it is crucial to use affordable technologies that can provide quick results at the point of care for early diagnosis. Biosensors that target specific biomarkers associated with different types of cancer offer an alternative diagnostic approach at the point of care. Recent advancements in manufacturing and design technologies have enabled the miniaturization and cost reduction of point-of-care devices, making them practical for diagnosing various cancer diseases. Furthermore, machine learning (ML) algorithms have been employed to analyze sensor data and extract valuable information through the use of statistical techniques. In this review paper, we provide details on how various machine learning algorithms contribute to the ongoing development of advanced data processing techniques for biosensors, which are continually emerging. We also provide information on the various technologies used in point-of-care cancer diagnostic biosensors, along with a comparison of the performance of different ML algorithms and sensing modalities in terms of classification accuracy.

## 1. Introduction

Cancer is a major cause of death worldwide, and the incidence of cancer continues to rise, making early detection and diagnosis essential. In 2020, nearly 10 million people died of cancer, according to the World Health Organization [1]. Cancer is a genetic disease caused by changes in the DNA sequence of cells due to DNA damage, harmful substances, or errors in cell division [2]. Lung and breast cancers are prevalent types of cancer that affect men and women. Carcinoma, lymphoma, leukemia, sarcoma, and melanoma are frequently encountered cancer classifications that can emerge in various organs of the human body. Aging, prolonged exposure to sunlight, smoking, radiation exposure, viral infections, the use of hormonal medications, and exposure to certain chemicals are among the known factors contributing to the development of cancer [3].

Cancer involves the abnormal growth of cells that are clones of each other, and these cancerous cells can divide and spread beyond the neighboring cells, generating tumors. Tumors can be benign or malignant [4]. Benign tumors stay in their primary stage and may grow in size without spreading into neighboring tissues or organs. In contrast, malignant tumors consist of cancer cells that grow uncontrollably and spread into neighboring cells, tissues, or organs. Cancer cells can spread to other parts of the body via the lymphatic node system or through the bloodstream. From benign tumors to malignant, cancer passes through four stages, where the fourth stage is called metastatic, and the chances of survival are very low [5]. Unfortunately, there are no advanced methods to diagnose cancer at the earliest stages.

Typical cancer detection processes involve physical exams, lab tests that use expensive and harmful equipment based on electromagnetic radiation, or a surgical diagnosis called a biopsy [6]. These lab tests use biological samples, such as blood, urine, saliva, or sweat, to detect specific biomarkers. Imaging-based tests include magnetic resonance imaging (MRI), computed tomography (CT), positron emission tomography (PET), ultrasound, and X-rays [7,8,9,10]. Although these imaging tests are non-invasive, they require expensive equipment and experienced pathologists. Biopsies are useful for determining the type of tumor, but they are invasive, painful, time-consuming, and expensive [11,12,13]. These issues with the current cancer diagnostic and detection methods make them unsuitable for point-of-care testing (POCT) and early cancer diagnosis [14,15,16,17,18,19]. Therefore, there is an urgent need to develop biosensing platforms for early cancer diagnosis that are low-cost, non-invasive, accurate, less time-consuming, and can be used at the point of care.

Biosensors can fill the void of point-of-care testing by providing low-cost, non-invasive, high-speed, and on-site testing of bio samples. A biosensor is a specialized type of sensor designed to detect and capture biological signals or molecules within a given sample or environment. These biological signals can include various entities, such as specific proteins, enzymes, DNA sequences, antibodies, or even whole cells. Biosensors are particularly significant in the field of medical and healthcare applications due to their ability to provide valuable information about biological processes, disease diagnosis, and patient monitoring [20]. Various point-of-care devices and lab-on-chip technologies have been employed to detect and diagnose various biomarkers from bio samples, such as pregnancy tests, blood glucose, or tuberculosis. Biosensors search for a specific biomarker in the bio sample and use either electrical or optical sensing modalities to detect biomarkers [19,21,22,23,24,25,26] associated with a cancer type. Additionally, researchers have conducted significant research on biosensors that use sweat or exhaled air as a potential sensing mode and have tried to associate the concentrations of volatile organic compounds with the presence of cancer or some other disease [25,27]. 

Biosensors can generate large amounts of data at the time of diagnosis and may require complex processing algorithms to generate the results. To effectively analyze the vast amounts of data obtained from biosensors and extract valuable information, researchers have harnessed the power of machine learning (ML) techniques. These methods include but are not limited to support vector machines (SVM), k-nearest neighbor (KNN), decision trees (DT), artificial neural networks (ANN), and convolutional neural networks (CNN) [28,29]. This paper is a review of the research projects that develop biosensors for early cancer detection and diagnosis and employ ML algorithms to enhance data analysis. In this regard, a review of ML algorithms is presented, along with the fundamental working principles of different biosensing techniques. The papers in this review study are divided into two main categories: electrical detection and optical detection biosensors. To facilitate a reader’s understanding of how ML models are advancing biosensors in diagnosing different types of cancer, sub-categories have been created based on the type of cancer detected using specific biomarkers or cells.

## 2. Overview of Machine Learning Algorithms

Machine learning (ML) has grown rapidly over the past few decades and has widely used applications not only limited to healthcare problems, such as predicting drug discoveries and diagnosing diseases, but also in other fields, such as mechanics, robotics, and image recognition [30,31,32,33,34]. In simple words, ML is a rapidly developing field of computational algorithms that aims to replicate human intelligence by adapting to their surroundings and learning from them [35]. There are two main types of machine learning algorithms: supervised and unsupervised learning [36]. The difference between these two main classes is the existence of labels in the training data subset, which will be discussed in the following sections.

### 2.1. Supervised Machine Learning

Supervised algorithms are a subset of machine learning models which generate a function that maps inputs to desired outputs [37]. Supervised learning is characterized by the usage of labeled datasets to train algorithms for accurate classification or outcome prediction. The model adjusts its weights as input data is fed into it, achieving proper fitting during the cross-validation process [38]. During the model training process, the predicted output is compared to the actual output, and modifications are made to decrease the overall error between the two. Supervised machine learning algorithms have a broad range of applications in biosensors and healthcare, including tasks such as distinguishing cancer from non-cancer cells, detecting circulating tumor cells (CTCs), and predicting DNA quantities [31,38,39]. In the following sections, the most well-known and commonly supervised algorithms will be discussed.

#### 2.1.1. Support Vector Machines (SVMs)

**Figure 1:**
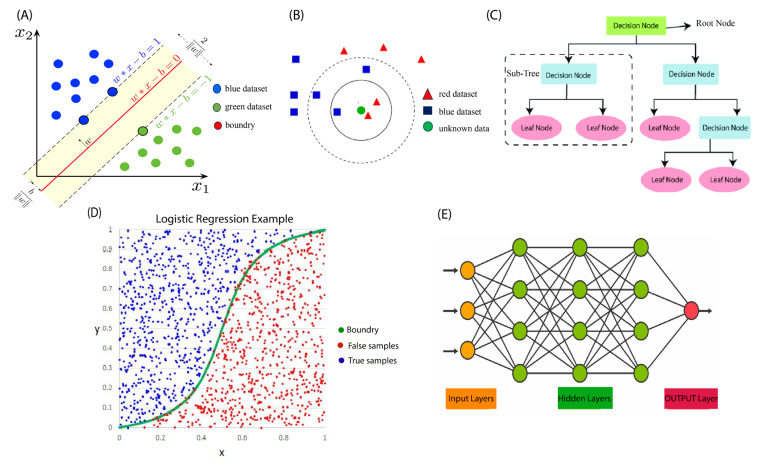
Schematic illustrations of supervised machine learning algorithms. (**A**) SVM model. Reprinted from [52]. (**B**) KNN model Reprinted from [53]. (**C**) DT model. Reprinted from [54]. (**D**) LR model Reprinted from [55]. (**E**) ANN model. Reprinted from [56].

The support vector machine algorithm is a popular supervised algorithm used both in classification and regression models [40]. In classification, the SVM aims to identify a hyperplane in an N-dimensional feature space, which effectively separates the data points into distinct classes (Figure 1A), while, in regression models, the SVM aims to find a line that best fits the data [41]. The kernel-based SVM algorithm uses kernel functions to transform the input data into a higher dimensional feature space when the data cannot be separated linearly. The performance of the SVM model depends on two hyperparameters: kernel parameters and kernel types. The selection of the kernel type is determined based on the characteristics of the input data [29].

#### 2.1.2. K-Nearest Neighbor (KNN)

The k-nearest neighbor (KNN) algorithm is a type of supervised machine learning algorithm that classifies objects based on the classes of their nearest neighbors [42]. It is typically used for classification but can also be applied to regression problems. The algorithm predicts the class or value of a new data point based on the k-closest data points in the training dataset. To identify the nearest neighbors, the algorithm calculates the distance between the new data point and all other data points in the dataset. For example, in Figure 1B, the green unknown data point belongs to the red dataset. For classification, the algorithm assigns the new data point to the most common class among its k-nearest neighbors, while for regression analysis, it calculates the average value of the k-nearest neighbors and assigns it to the new data point [42]. The value of k is usually determined through cross-validation or other optimization techniques, and it impacts the bias-variance trade-off of the model. Despite its simplicity, KNN is a highly effective algorithm and is widely used in many fields, including image recognition, natural language processing, and healthcare problems [43,44,45].

#### 2.1.3. Decision Tree (DT)

The decision tree algorithm is a popular supervised machine learning algorithm used for classification and regression tasks. It works by constructing a tree-like model of decisions and their possible consequences based on the data [46]. The decision tree algorithm works by dividing the feature space of the training set recursively. Its goal is to identify a collection of decision rules that can partition the feature space in a way that produces a reliable and informative hierarchical classification model. In this algorithm, each node represents an attribute or feature, and each branch represents an outcome. The root node represents the entire dataset, and at each internal node, the algorithm divides the data based on a specific attribute’s value. The schematic of the DT algorithm is shown in Figure 1C. This process is repeated recursively until a stopping condition is met, such as achieving a specified level of purity or reaching a predetermined depth [46].

#### 2.1.4. Gaussian Naïve Bayes (GNB)

The Gaussian naïve Bayes (GNB) algorithm is a classification technique used in machine learning that leverages a probabilistic approach and the Gaussian distribution to make predictions of input data. GNB treats each attribute variable as independent, enabling it to be trained efficiently in supervised learning and used in complex real-world scenarios. GNB is particularly effective when dealing with high-dimensional data since it assumes independence between features, making it less susceptible to the curse of dimensionality [47].

#### 2.1.5. Logistic Regression (LR)

Logistic regression is a supervised machine learning algorithm designed to solve classification problems where the target variable is categorical. The primary objective of logistic regression is to establish a mapping function from the dataset’s features to the target. This allows the algorithm to predict the probability of a new data point belonging to a particular class [48]. As shown in Figure 1D, the input space is divided into two regions, which are separated by a boundary. Logistic regression is a widely used algorithm in many fields, such as marketing, healthcare, and finance, as it can help identify patterns and relationships between variables that can assist in making accurate predictions and decisions [49].

#### 2.1.6. Random Forest (RF)

Random forest is a supervised machine learning algorithm that builds on the concept of tree classifiers. It generates a large number of classification trees and uses them to classify new feature vectors. Each tree in the forest classifies the input vector, and the tree’s classification is counted as a “vote” for that class. The forest then chooses the classification with the highest number of votes across all the trees in the forest as the final prediction. RF is a highly effective algorithm for handling complex, high-dimensional datasets. It uses ensemble learning to reduce overfitting and improve the model’s accuracy by combining the outputs of multiple decision trees [50].

#### 2.1.7. Artificial Neural Network (ANN)

Artificial neural networks (ANNs) are computer programs designed to mimic the way the human brain processes information. They derive their inspiration from biological neural networks and adopt a similar structure of interconnected neurons to perform complex tasks. ANNs acquire knowledge through experience by identifying patterns and relationships in data instead of relying on explicit programming to accomplish the task.

An ANN typically consists of many processing elements (PE), also known as artificial neurons, which are connected by weights. These weights constitute the neural structure of the network and are organized into layers. The structure of an ANN is shown in Figure 1E. Through a process of training, the network learns to adjust the weights between the neurons to produce the desired output given a specific input. ANNs can be used for a variety of tasks, such as image and speech recognition, natural language processing, predictive analytics, and healthcare [51]. Figure 1 represents common supervised ML algorithms.

### 2.2. Unsupervised Machine Learning

Unsupervised learning is a subfield of machine learning where the data provided to the machine learning algorithm is unlabeled, and it is up to the algorithm to make sense of the data on its own. In unsupervised learning, the algorithm looks for patterns and structures in the data and tries to group similar data points together based on their similarities or differences. One of the key advantages of unsupervised learning is that it can reveal insights and relationships that may not be immediately apparent to human observers. By discovering patterns and similarities in the data, unsupervised learning can help uncover the hidden relationships that can be useful for making decisions or solving problems. For example, unsupervised machine learning can be used to identify customer segments in a marketing dataset or to find anomalies or outliers in a dataset that may indicate fraudulent activity [41].

### 2.3. Machine Learning Figures of Merits

To evaluate the performance of the representative model, the following metrics are used: accuracy (ACC), true positive rate (TPR), true negative rate (TNR), false negative rate (FNR), and false positive rate (FPR). These measures are computed using the following forms:

(1) $$\mathrm{Accuracy}\left(\mathrm{ACC}\right)=\frac{\mathrm{TP}+\mathrm{TN}}{\mathrm{TN}+\mathrm{TP}+\mathrm{FN}+\mathrm{FP}}$$

(2) $$\mathrm{Sensitivity}\left(\mathrm{TRP}\right)=\frac{\mathrm{TP}}{\mathrm{TP}+\mathrm{FN}}$$

(3) $$\mathrm{Specificity}\left(\mathrm{TNR}\right)=\frac{\mathrm{TN}}{\mathrm{TN}+\mathrm{FP}}$$

(4) $$\mathrm{Fallout}\left(\mathrm{FPR}\right)=\frac{\mathrm{FP}}{\mathrm{TN}+\mathrm{FP}}$$

(5) $$\mathrm{False}\;\mathrm{Negative}\;\mathrm{Rate}\left(\mathrm{FNR}\right)=\frac{\mathrm{FN}}{\mathrm{TP}+\mathrm{FN}}$$

where the TP’s and FPs refer to the number of correct and incorrect predictions of outcomes to be in the considered output class, whereas the TN’s and FNs refer to the number of correct and incorrect predictions of outcomes to be in any other output classes respectively [30].

The ROC (receiver operating characteristic) curve is a graphical representation of the performance of a binary classification model. It is a graph that shows the trade-off between the true positive rate and the false positive rate. A diagonal line in the ROC curve indicates that the test has no discriminatory ability, while an ROC curve above the diagonal line indicates a test with better-than-chance discrimination ability. The area under the ROC curve (AUC) is a measure of the overall ability of the test to discriminate between the presence or absence of a condition. An AUC of 1.0 indicates perfect discrimination, and an AUC of 0.5 indicates no discriminatory ability [57].

## 3. Lab-on-a-Chip in Cancer Detection

This section provides a brief overview of the various types of sensors, including optical and electrical sensors, such as image-based, fluorescence, and impedance sensors, that can be used to detect cancer cells. Additionally, the results of machine learning algorithms to analyze data collected from these sensors have been discussed.

### 3.1. Optical Detection

Optical detection of cells implies the use of optical techniques and instruments for the detection, classification, and stratification of cells [41]. Various types of optical-based biosensors have been developed and utilized for diverse biological and clinical applications, such as surface plasmon resonance (SPR), optical waveguides, optical resonators, and fluorescence [3,58,59]. Optical biosensors have several advantages, including their high sensitivity, real-time detection, label-free analysis, small form factor, and low cost [41]. These characteristics make optical biosensors an appealing option for integration into lab-on-a-chip devices, which seek to carry out sample preparation, research, and detection in a miniaturized and automated format [59]. In the next section, we will review the latest developments of optical-based biosensor devices in the identification and clinical diagnosis of various types of cancers, as well as data analysis with machine learning techniques.

#### 3.1.1. Breast Cancer

Based on the World Health Organization (WHO), breast cancer is the most frequent cancer among women, affecting over 1.5 million women each year, and is responsible for the most significant cancer-related deaths among women. In 2015, 570,000 women died from breast cancer [60]. This highlights the potential of biosensors for the early detection of cancer cells. Biosensors are promising and selective detection devices which hold immense potential as point-of-care (POC) tools [61]. Several studies have shown the application of optical-based biosensors to detect breast cancer cells, demonstrating the promising potential of biosensors for early-stage detection of breast cancer cells.

**Figure 2:**
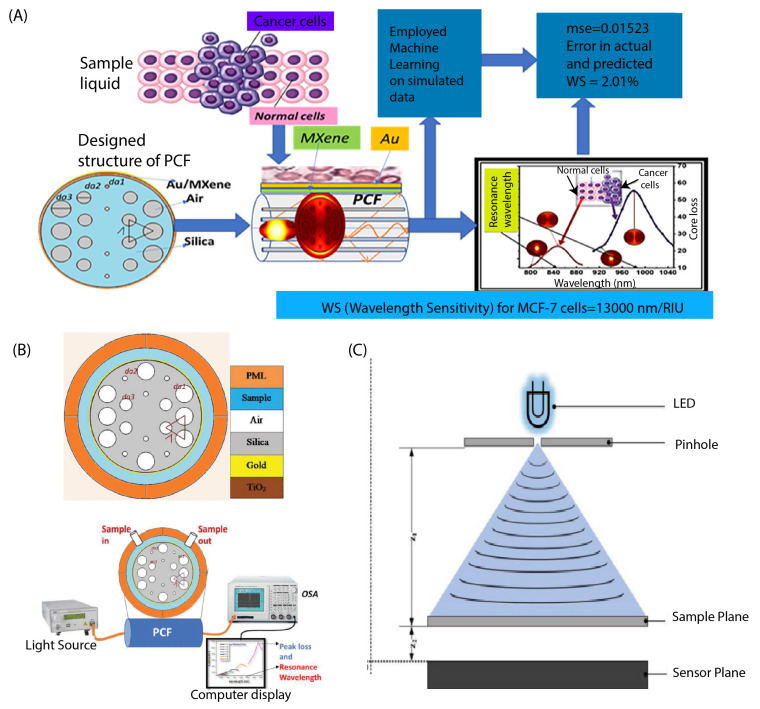
(**A**) Cross-sectional view of the proposed SPR sensor with the experimental setup. Reprinted with permission from [64]. Copyright 2023 Elsevier. (**B**) Colored entities of the designed sensor with a cross-sectional view with the experimental setup. Reprinted with permission from [65]. Copyright 2023 IEEE. (**C**) Schemes of the digital in-line holographic microscope (DIHM). Reprinted with permission from [68]. Copyright 2023 Springer Nature.

The surface plasmon resonance (SPR) sensor is an optical sensor employing a unique mode of electromagnetic field called the surface plasmon, which propagates at the interface of a metal and a dielectric. The SPR sensor utilizes the evanescent field generated by the surface plasmon to detect alterations in the refractive index of the dielectric material near the interface [62]. Numerous studies have suggested the effectiveness of SPR sensors in the early detection of cancers [63]. Kumar et al. [64] described a photonic crystal fiber-based surface plasmon resonance (SPR) sensor for detecting breast cancer cells based on their refractive index (Figure 2A). They used simulations and numerical analysis to measure the wavelength sensitivity and resolution of the sensors for normal and cancerous cells, achieving a high sensitivity and low resolution. The refractive index of normal and cancerous cells was estimated using a multi-layer perceptron, and the machine learning algorithm was used to optimize the structural parameters. The proposed sensor shows promising results and could be a potential alternative sensing device for early-stage breast cancer diagnosis. In another study, Verma et al. [65] developed a machine learning approach for breast cancer cell detection using a surface plasmon resonance (SPR) based on a photonic crystal fiber sensor, which is shown in Figure 2B. The sensor operates by detecting changes in the refractive index of the fiber when breast cancer cells are present. The machine learning algorithm is trained on a dataset of SPR spectra obtained from both breast cancer and non-cancerous cells and is used to classify new samples as either cancerous or non-cancerous based on their spectral patterns.

Another type of optical sensor is the fluorescence sensor, widely used to identify and measure biomolecules or metal ions. The advantages of this type of sensor include its sensitivity, specificity, resistance to light scattering, and ease of use [66]. In a study reported by Jin et al. [67], they developed a breast cancer liquid biopsy system that integrates a fluorescence sensor array with a deep learning model. The sensor array uses fluorescent probes to gather diverse information about cells and exosomes. The deep learning model employs a CNN-based architecture to distinguish between normal and cancerous cells. The system has demonstrated successful discrimination between normal and different cancerous cells and achieved a 100% accurate classification of different breast cancer cells. In addition, Pala et al. [68] constructed and tested a digital in-line holographic microscope for imaging breast cancer cells using holography, which is shown in Figure 2C. The microscope was constructed using a white LED for illumination, a pinhole to make the light semi-coherent, and a CMOS sensor to record images of the plane above it. Holograms were captured and numerically reconstructed, and the amplitude of individual cells was collected. Using machine learning, these images were transformed into a fractal dimension and rotated to calculate the identifying features of each cell. Upon testing the accuracy of this system, the team achieved an accuracy of 99.65%.

#### 3.1.2. Lung Cancer

Lung cancer is a primary cancer that poses a significant threat to human life globally, having the highest mortality rate. Early detection of lung cancer is crucial for a timely diagnosis and subsequent treatment. However, conventional methods for lung cancer detection have limitations, such as their low sensitivity, high costs, and invasive procedures, which restrict their practicality [69]. In this section, we will review the optical biosensors for detecting lung cancer and explore how machine learning can aid in analyzing data and improving their application.

**Figure 3:**
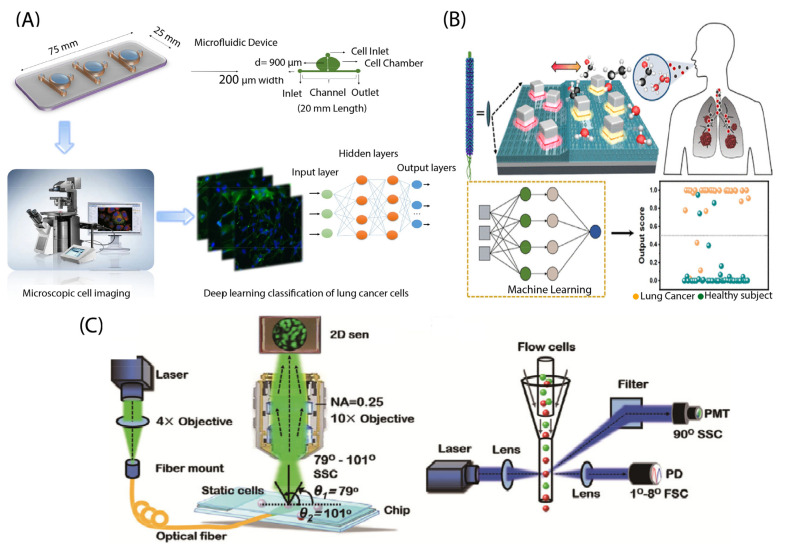
(**A**) Overview of the combined microscopic cell imaging and deep learning approach. Reprinted from [70]. Copyright 2021 Springer Nature. (**B**) Schematic of the biosensor with the combination of machine learning methods to detect lung cancers. Reprinted from [72]. (**C**) Schematic of the experimental setups. Reprinted with permission from [73]. Copyright 2018 John Wiley and Sons.

Image-based detection implies the use of images or videos of cells. These images or videos need to be processed to identify and quantify cells [41]. To demonstrate, Hashemzadeh et al. [70] developed a microfluidic chip for lung cancer detection that employs image-based analysis. Images were obtained using an inverted Olympus fluorescence microscope and were analyzed by a deep learning model. The researchers achieved an accuracy of 98.37% in classifying images of lung cancer cell lines and normal cell lines. The overview of the combined microfluidic deep learning approach has been shown in Figure 3A. As another example, Sui et al. [71] described the development of a microfluidic imaging flow cytometer that can detect lung cancer using complex-field imaging and fluorescence detection subsystems. The system can analyze millions of cells and provide a hierarchical analysis of the intrinsic morphological descriptors of single-cell optical and mass density, as well as fluorescently labeled biochemical markers. The data collected from the system were used to train deep learning-based models, which achieved a classification accuracy ranging from 91% to 95% for lung cancer detection.

Volatile organic compounds (VOCs) are potential biomarkers for lung cancer detection. In the study reported by Nguyen et al. [72], a controllable gap plasmonic color film biosensor was developed for the detection and quantification of VOCs. The goal of the study was to diagnose lung cancer based on VOC gas detection from exhaled breath samples. The color changes in the sensor arrays when exposed to humidity and VOCs were recorded using a camera, and a CNN model was trained to classify them into different VOCs (Figure 3B). They collected the data from 70 healthy and 50 lung cancer patients and trained the ML models, reporting a training classification accuracy of 90% and 92.8% for lung cancer and healthy patients, respectively. They achieved a classification accuracy of 89% on the test data.

A label-free classification of lung cancer cell lines was developed by Wei et al. [73] by using a two-dimensional (2D) light-scattering static cytometric technique. In this study, a method for the automatic classification of small cell lung cancer (SCLC) and poorly differentiated lung adenocarcinoma (PD-LUAD) cells was introduced by using 2D light-scattering static cytometry and machine learning (Figure 3C). A laser was used to detect the cells by means of a two-dimensional light-scattering static cytometric technique, where measurements of forward and side scattered light enabled the differentiation of overlapping SCLC and PD-LUAD cells. By employing a support vector machine (SVM) classifier, the team achieved the classification of these cells with an accuracy greater than 99.78%.

Feature extraction plays a vital role in machine learning when dealing with large amounts of data. It helps to identify and extract the most relevant and informative aspects or characteristics from the data, enabling a more effective and efficient analysis [74]. For example, Ahmad et al. [75] presented a microfluidic platform and light-sheet fluorescence microscopy based on a single-cell classification system to classify human mammary epithelial cells, primary tumor cells, and lung metastasis-derived cells. They used an optofluidic device to deliver single cells to the fluorescent microscope and simulated 3D point clouds of the fluorescent markers. They applied feature extraction techniques along with custom CNN models to classify the images. The authors achieved high accuracy on both the simulated and actual datasets and studied the effects of varying flow rates on accuracy. They reported an accuracy of 99.4% on the actual dataset.

**Figure 4:**
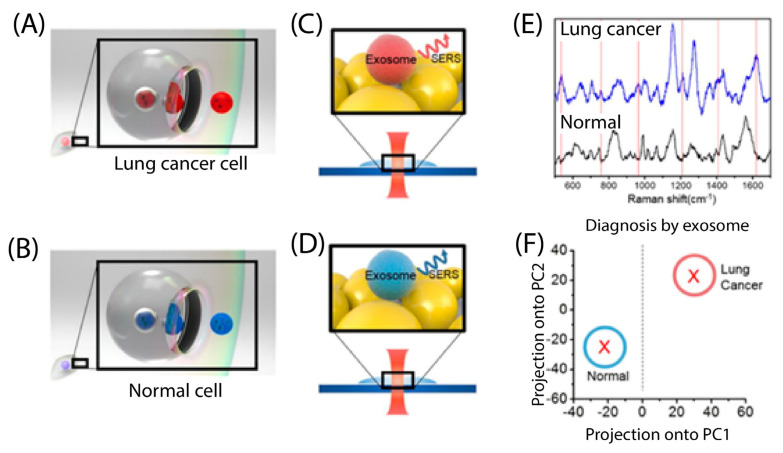
Schematic diagram of lung cancer diagnosis by SERS classification of the exosome. (**A**,**B**) Lung cancer cells and normal cells release exosomes to the extracellular environment, having their own profiles by fusing multivesicular endosomes to the plasma membrane, respectively. (**C**,**D**) Raman spectra of lung cancer cells and normal cell-derived exosomes were achieved by SERS, respectively. (**E**) SERS spectra, achieved by methods of panels (**C**,**D**), are shown. Red lines indicate specific peaks of lung cancer-derived exosomes. (**F**) Exosome classification is obtained by PCA of SERS spectra. Reprinted with permission from [77]. Copyright 2017 American Chemical Society.

Surface-enhanced Raman scattering (SERS) is a powerful method for identifying chemical information at a single molecular scale [76]. Lin et al. [76] developed a new biosensing platform that can identify and differentiate exosomes derived from cancerous and non-cancerous sources. The platform uses a porous-plasmonic SERS chip with CP05 polypeptide to capture and distinguish exosomes without the need for labeling or purification. By combining biological analysis with Raman spectra and machine learning methods, the team accurately differentiated between lung and colon cancer cell–derived exosomes and normal exosomes at the single vesicle level, achieving an 85.72% accuracy. This protocol is fast, reliable, and easy to operate, making it a promising tool for early tumor detection and prognosis. As another example, Park et al. [77] used SERS and statistical pattern analysis to identify lung cancer cells (Figure 4). Instead of looking at specific peak positions and amplitudes in the spectrum, they analyzed the whole SERS spectra of exosomes using principal component analysis (PCA). Using this approach, they were able to distinguish the exosomes derived from lung cancer cells from those derived from normal cells, with a 95.3% sensitivity and 97.3% specificity.

#### 3.1.3. Gastrointestinal Cancer

In this section, we will review the application of optical-based biosensors in combination with machine learning to analyze the data collected from sensors for the detection of gastrointestinal cancers, such as pancreatic and liver cancers.

Nowadays, many studies detect pancreatic cancer cells using exosomes, which are small vesicles secreted by cancer cells, as biomarkers for detection. To demonstrate, Ko et al. [78] developed a multichannel nanofluidic system to analyze crude clinical samples. They used exosomes as biomarkers for detecting pancreatic cancer. The exosomes were isolated and analyzed using a microfluidic chip with a nanoporous membrane that allowed the capture of exosomes based on their size. The captured exosomes were then analyzed using machine learning algorithms to classify them as either cancerous or non-cancerous. The results showed that the approach had a high accuracy with an area under curve (AUC) of 0.81 in diagnosing pancreatic cancer, indicating its potential for use in clinical settings as a non-invasive diagnostic tool. As another example, Li et al. [79] developed a new method for detecting colorectal cancer using exosomes as a specific protein biomarker. They created a microfluidic chip with a 3D porous sponge structure and functionalized it with CD9 antibodies to capture exosomes flowing through the microfluidic channel. The authors then used an anti-SORL1 antibody modified with Si-QD silicon quantum dots to label the captured exosomes and obtain fluorescence images. They extracted three features (luminance, mean, and variance) and trained an RF algorithm to classify the exosomes. The authors report that they achieved a high classification accuracy of 91.14%. Last but not least, Cheng et al. [80] described a nano biosensing chip that utilizes SERS to detect cancer without the need for antibodies. This study showcased a simple and intelligent detection method for efficiently screening liver cancer, achieving a sensitivity of 90% and specificity of 92% in identifying 50 serum SERS spectra from HCC patients compared to 50 serum SERS spectra from healthy individuals.

D’Orazio et al. [81] introduced the concept of machine learning phenomics (MLP), which combines deep learning with time-lapse microscopy to monitor drug responses in colorectal cancer cells. This study aims to evaluate the effectiveness of this approach by comparing it with the conventional methods used to analyze drug responses in these cells. The results demonstrate that MLP can accurately predict drug responses in colorectal adenocarcinoma cells based on their gene expression patterns, and it outperforms the conventional methods in terms of accuracy and efficiency.

Quantum dot immunobionsensors are powerful optical sensors used to detect cancer cells, which were introduced by Saren et al. [82] to detect and quantify gastrointestinal tumor biomarkers. They developed quantum dot (QD)-labeled biofilms to detect four biomarkers: CEA, CA125, CA19-9, and AFP, indicating the presence of gastrointestinal tumors. The antibody conjugates of the QD were analyzed using fluorescence and ultraviolet absorption spectroscopy. The PCA technique was applied to the images obtained from the data collected. The approach was tested on standard samples rather than clinical samples, achieving a classification precision of 99.52% and 99.03% and classification accuracy of 94.86% and 94.2% for colon tumors and gastric tumors, respectively.

Pyruvate kinase disease (PKD) is an inherited disorder that affects red blood cell metabolism and may have an increased risk of developing liver cancer and some types of colon and kidney cancer [83,84]. Mencattini et al. [85] described a machine learning microfluidic-based platform that integrates lab-on-chip devices and data analysis algorithms to evaluate the plasticity of red blood cells in PKD monitoring. The platform uses microfluidic channels to measure the deformability of red blood cells, which is a critical indicator of the disease. The data collected from the microfluidic device are then analyzed using machine learning algorithms to determine the severity of the disease. The blood cells were recorded through a ‘forest of pillars’, and the video was saved for offline analysis. The efficacy of three networks, AlexNet, ResNet-101, and NasNetLarge, pre-trained deep learning architectures, was tested on actual samples. On the live samples, the performance of AlexNet was 88%, ResNet-101 was 82%, and NasNetLarge was 85%.

#### 3.1.4. Gynecological Cancer

The most common types of gynecological cancers are cervical, ovarian, and endometrial (uterine) cancers. Late diagnosis and chemoresistance present significant obstacles to the successful treatment of gynecological cancers. Therefore, there is a pressing need to develop new markers to detect gynecological cancers at an early stage. In this regard, biosensors that are low-cost and non-invasive hold great potential for predicting these types of cancers at an early stage [86]. Moreover, with the emergence of biosensors that generate large amounts of data, the application of machine learning to analyze this data has become increasingly important.

**Figure 5:**
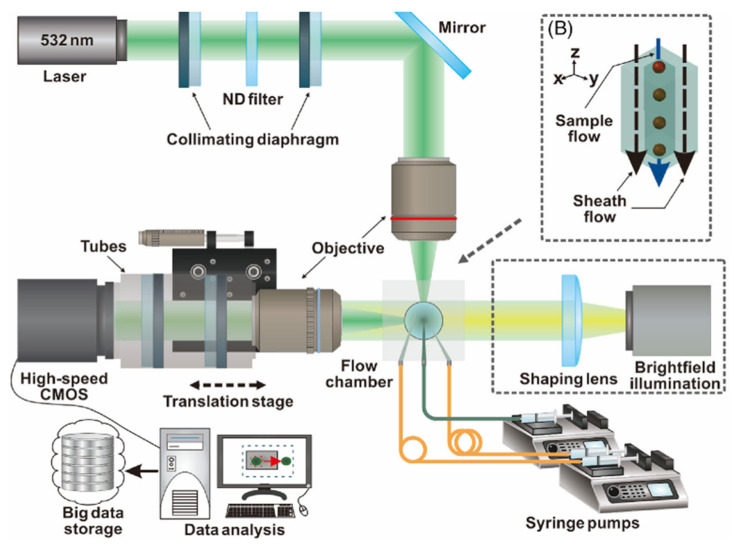
The schematic diagram of high-content VFC and the 3D schematic diagram of the sheath flow in the flow chamber. Reprinted with permission from [87]. Copyright 2022 John Wiley and Sons.

High-content VFC (video flow cytometry) utilizes a 2D light-scattering technique to project optical signals from cells onto an image sensor without optical focusing. This allows for high-content patterns to be obtained and combined with machine learning algorithms, enabling automated, high-throughput analysis of single cells. The VFC technique developed by Liu et al. [87] achieves a measurement rate of around 1000 unlabeled cells per minute and demonstrates high accuracy in classifying cervical carcinoma cell lines, including Caski, HeLa, and C33-A cells. An accuracy of 91.5%, 90.5%, and 90.5% for these cell lines by using a deep learning model has been reported. This study provides high-quality cell images, automatic digital filtering, and label-free cell classification, offering potential clinical applications. The illustration of our high-content VFC is shown in Figure 5.

Serum biomarkers are frequently utilized due to their sensitivity and specificity, which makes them valuable for cancer screening or diagnostic testing purposes. To demonstrate, Kim et al. [88] developed a nanosensor array and a computational model that resulted in the perception-based detection of ovarian cancer from patient serum samples. The researchers aimed to develop a novel approach for diagnosing ovarian cancer based on the unique spectral characteristics of carbon nanotubes modified with quantum defects. They utilized machine learning algorithms to analyze the spectral data obtained from the serum samples. They trained and validated several machine learning classifiers with 269 serum samples to distinguish patients from those with other diseases and healthy individuals. Their results showed that the SVM algorithm yielded the best F-scores among the five machine learning algorithms tested, with an accuracy of 95%.

In another study reported by Pirone et al. [89], a digital holography to model cells in 3D space instead of 2D space was developed. This method provides a better characterization of endometrial cancer cells. They extracted 67 features, such as morphology and histogram, as inputs of machine learning algorithms. In order to test the classification performance with the 3D and 2D features, several common machine-learning methods have been trained and tested on the feature data. The results show that 3D features achieve a better classification performance, and the LDA classifier achieves the best score.

#### 3.1.5. Prostate Cancer

Prostate cancer (PCa) is a widespread health concern, affecting 1.3 million men globally in 2018 [90]. Detecting prostate cancer (Pca) in its early stages is vital for effective treatment, and the utilization of biosensors can assist in the early detection of Pca. Furthermore, by utilizing machine learning to analyze the vast amounts of data generated by biosensors, we can achieve highly accurate predictions, ultimately leading to better patient outcomes.

Prostate cancer gene 3 (PCA3) is specifically expressed in the prostate and is strongly associated with prostate cancer. It shows promise as a potential biomarker for detecting prostate cancer. The PCA3 gene has been detected in 95% of prostate cancer samples, making it highly associated with the disease. Even small amounts of PCA3 can indicate a significant likelihood that a patient either has or will develop prostate cancer [91]. As an example, Rodrigues et al. [91] developed a genosensor with carbon-printed electrodes and a layer of a complementary DNA sequence (PCA3 probe). They investigated the ability of electrochemical and optical detection methods, along with machine learning algorithms, to diagnose prostate cancer using images of the genosensors. The study demonstrated that the meta-classifier machine learning algorithms, including SVM and LDA, could accurately classify scanning electron microscopy images with 99.9% accuracy.

Differences in the metabolite components between patient urine and normal urine have been reported, indicating the need for a rapid, easy-to-use, and label-free technique to analyze urine metabolites. Such a technique is crucial for developing on-site urine diagnostic platforms and identifying unknown metabolite biomarkers for cancer detection. In a study conducted by Ling et al. [92], the researchers applied an integrated on-site detection system based on SERS sensor technology and deep learning models to diagnose prostate and pancreatic cancer. The sensor is based on a 3D plasmonic coral nanoarchitecture (3D-PCN) synthesized on a paper substrate, which was integrated with a handheld Raman spectrometer to create an on-site diagnostic platform. Human urine samples are directly absorbed into the paper-based 3D-PCN, and the SERS signals of complicated urine components are obtained without any pretreatment. The RNN and CNN models are employed for the supervised classification of SERS spectra, and the platform achieved high sensitivity and specificity for detecting cancer. The system demonstrates the potential for use as a diagnostic platform in various human biofluid analyses in the future.

The bio-nanochip platform shows promising potential as a versatile and efficient biosensor system for various applications, including medical diagnostics and environmental monitoring. For instance, McRae et al. [93] designed a programmable bio-nanochip (p-BNC) system, a biosensor platform with the capacity for learning. In this system, small quantities of patient samples generate an immunofluorescent signal on agarose bead sensors that are optically extracted and converted to antigen concentrations. This biochip sensor has the potential to detect prostate cancer and ovarian cancer with single-use disposable cartridges. They applied machine learning methods to analyze the dataset.

#### 3.1.6. Brain Cancer

In this section, we will review the application of optical-based biosensors and machine learning for brain cancer detection.

**Figure 6:**
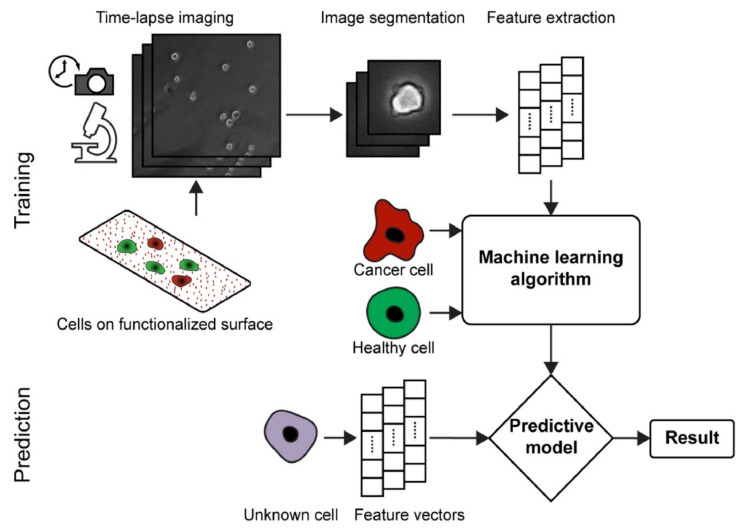
The schematic shows an overview of the dynamic morphological analysis of cell gestures. Reprinted with permission from [94]. Copyright 2018 Elsevier.

A supervised machine learning approach is commonly used for the identification and classification of cancer cell gestures, enabling early diagnosis. Hasan et al. [94] developed a system that captures time-lapse images of cancer cells and analyzes their morphological changes over time using image-processing techniques (Figure 6). The system also incorporates machine learning algorithms for the automated classification of cancer cells based on their dynamic morphology. As a proof of concept, the morphologies of human glioblastoma (hGBM), which causes brain tumors [95,96], and astrocyte cells were used. The cells were captured and imaged with an optical microscope. Three different classifier models, the SVM, RF, and naïve Bayes classifier (NBC), were trained with the known dataset using machine learning algorithms. All the classifier models detected the cancer cells with an average accuracy of at least 82%.

In another study, Hossain et al. [97] employed a sensor-based portable microwave brain imaging (SMBI) system to obtain the reconstructed microwave (RMW) brain images. The proposed method consists of a segmentation model called MicrowaveSegNet (MsegNet) and a classifier called BrainImageNet (BINet). A dataset of 300 RMW brain image samples was used to create an original dataset, which was then augmented to make 6000 training images for a five-fold cross-validation. The performance of MsegNet and BINet was compared to state-of-the-art segmentation and classification models, and the proposed models achieved impressive results. The proposed cascaded model has the potential to be used in sensor-based SMBI systems to investigate the progression of brain cancer disease.

#### 3.1.7. Hematological Cancer

Leukemic diseases are a diverse group of neoplasms that result from genetic disorders affecting hematopoietic precursor cells, and they represent one of the most common forms of hematologic cancer globally. Accurate diagnosis of these disorders requires specialized expertise and often involves using multiple techniques [98]. In this section, we presented the use of optical biosensors in conjunction with machine learning for detecting hematological cancer.

DNA methylation is a process in which a methyl group is attached to the fifth carbon atom of a cytosine (C) residue, resulting in the formation of 5-methylcytosine (5-mC). The methylation patterns in cancer genomes exhibit unique characteristics, known as the methyl cape, and can serve as a potential universal biomarker for cancer detection [99]. For example, Koowattanasuchat et al. [99] presented the development of a methyl cape sensing platform for leukemia screening using cysteamine-decorated gold nanoparticles (Cyst/AuNPs). The platform is based on methylation-dependent DNA solvation, and normal and cancerous DNAs have distinct methylation profiles. The authors report 95.3% accuracy in leukemia screening using an optical spectrophotometer and 90% accuracy when a smartphone system is used.

Minimal residual disease (MRD) testing is used mostly for blood cancers, such as lymphoma and leukemia [100,101]. Uslu et al. [102] investigated the signal readout mechanism of a biochip designed to detect MRD, which refers to highly resistant cancer cells that can cause relapse in cancer survivors after treatment. To improve the capture, isolation, and counting of these tumor cells, the team combined previously explored methods with the use of immunomagnetic beads. These beads are coated with receptors that bind to and capture target molecules, allowing them to be manipulated using magnetic fields. Once the unbound beads were filtered out of the microfluidic channel, the remaining beads were imaged at 20× and 40× magnifications using a CCD camera and processed using computer vision. The authors demonstrated the accuracy and reproducibility of the method through various experiments and comparisons with manual counting. They also discussed the potential applications of the automated method in research and clinical settings for the detection and monitoring of leukemia and other diseases. Machine learning algorithms to analyze the images obtained were utilized. Among the algorithms tested, the RF algorithm achieved the highest accuracy of 87.4%.

Tremendous progress has been made in the field of cancer treatment through the utilization of high-affinity T-cell receptors and chimeric antigen receptor (CAR)-modified T cells. These innovative approaches have recently obtained approval from the Food and Drug Administration (FDA) for treating certain hematologic malignancies [103]. To demonstrate, Sarkar et al. [103] implemented the droplet microfluidics-based cytotoxicity imaging approach to isolate individual natural killer cells. They measured their ability to kill cancer cells in the presence of different types of antibodies. Machine learning algorithms for analyzing the resulting data were used, and they predicted which types of antibodies were most effective in activating the natural killer cells.

Last but not least, Li et al. [104] presented a novel approach to improving the accuracy of blood cancer cells and biomarker identification in label-free flow cytometry using parallel quantitative phase imaging. Such technology holds promise for the early detection of primary cancer or metastasis. The team used this imaging technique to assess additional parameters, such as cell protein concentration, allowing for increased accuracy in categorizing unlabeled cells. Additionally, they developed a CNN that directly operated on the measurement signals of this setup to detect cancer cells more efficiently. They demonstrated the applicability of the new method in the classification of white blood cells and epithelial cancer cells with more than 95% accuracy in a label-free fashion. Table 1 provides a summary of the cancer cell types that were detected using optical biosensors, along with the outcomes of the machine learning algorithms applied to the data.

### 3.2. Electrical Detection

**Figure 7:**
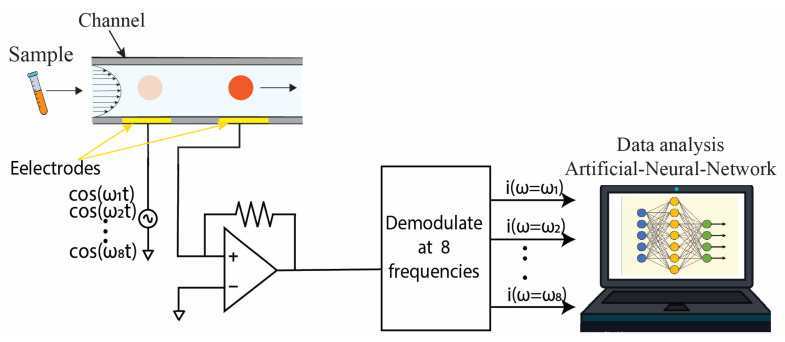
Schematic diagram of an electrical impedance cytometer. As cells flow through microfluidic chips, the change in impedance is measured by a lock-in amplifier. The lock-in amplifier can apply signals in different frequencies at a time. The data is then recorded and analyzed using the ANN algorithm.

The use of electrical circuits to gather data in the form of electrical signals is known as electrical detection. These signals can take the form of impedance, voltage, current, or any other electrical signal [41]. Among these, impedance is the most commonly used parameter for identifying and quantifying cells. As a cell or particle passes through the electrodes in a microfluidic channel, it causes a change in impedance, and the output signal is determined by the cell’s properties, such as size, conductivity, and permittivity. Compared to traditional optical detection, the electrical detection of cells has several advantages, including a smaller footprint and lower cost due to the absence of bulky optical equipment [41]. In the following paragraphs, we will discuss the biosensors that utilize machine learning techniques for the electrical detection of various cancer cells. A schematic diagram of an electrical impedance cytometer with ANN for data analysis is shown in Figure 7.

Within the preceding section, we provided an overview of the optical biosensors, which, in conjunction with machine learning, are utilized to analyze the data acquired from the sensors. Additionally, we emphasized the utility of an affordable and non-invasive biosensor in the detection of cancer cells. This section will focus on the employment of electrical-based biosensors in combination with machine learning for the detection of cancer.

#### 3.2.1. Breast Cancer

**Figure 8:**
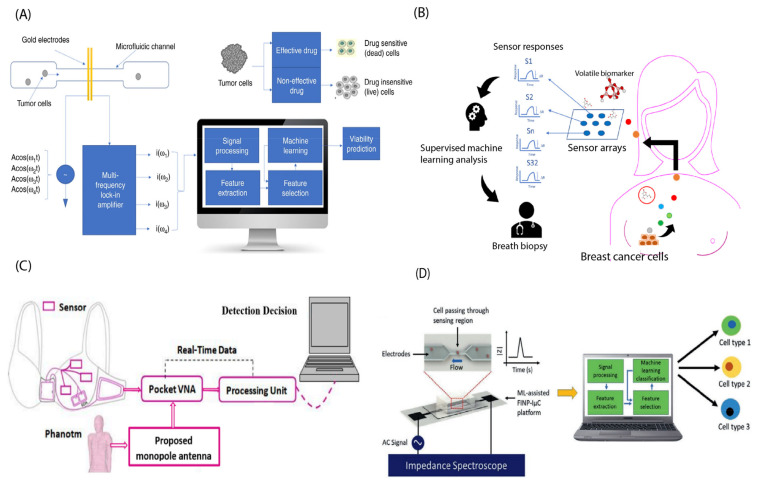
(**A**) Multifrequency impedance cytometry measures the response across a broad range of frequencies to assess cellular responses to a target drug. Machine learning algorithms are utilized to predict the viability of both live and dead cells. Reprinted with permission from [105]. Copyright 2021 Springer Nature. (**B**) Graphical representation illustrating the concept of breath biopsy. Breast cancer cells produce volatile metabolites that travel to the lungs and are exhaled. By using a sensor array to analyze these biomarkers in the breath, we can identify the molecular subtype of breast cancer at an early stage. Reprinted from [107]. (**C**) The proposed breast cancer detection system is a Smart Bra. Reprinted with permission from [108]. Copyright 2020 John Wiley and Sons. (**D**) The ML-assisted biochip performs single-cell classification in a label-free manner. The machine learning algorithm is used to perform both cell health classification (cancerous vs. non-cancerous) and cancer subtype cell discrimination at the single cell level. Reprinted with the permission from [109]. Copyright 2020 John Wiley and Sons.

Electrical impedance spectroscopy/cytometry is a technique that allows the measurement of AC electrical properties of particles in a liquid suspension. This method provides information about the frequency-dependent dielectric parameters of the particles. The main advantage of impedance cytometry is its label-free nature, allowing analysis to be conducted at the individual cell level [41]. To demonstrate, a study by Ahuja et al. [105] presented a microfluidic device that utilizes multifrequency impedance spectroscopy and supervised machine learning, which is shown in Figure 8A, to rapidly evaluate the tumor cell’s sensitivity to drugs. In this experiment, T47D cancer cells, which are a type of breast cancer cell, were passed through a microfluidic chip and their impedance and phase features were recorded. The goal of this experiment was to classify T47D cancer cells treated with the target drug and T47D dead cancer cells. The resulting classifier exhibited an accuracy of 95.9% using amplitude change and phase change as features for the SVM classifier.

A surface acoustic wave (SAW) biosensor is an electrical biosensor. It operates based on the generation and detection of surface acoustic waves on a piezoelectric substrate, which are electrical signals. Sountharrajan et al. [106] developed a SAW biosensor for the label-free detection of HER-2/neu, a biomarker associated with breast cancer cells. The biosensor output, along with data from the Wisconsin dataset (the name of the breast cancer dataset), was inputted into a proposed system for data mining classification algorithms. The proposed model was improved by ranking the attributes using the Ranker algorithm, resulting in an accuracy of 79.25% using an SVM classifier. Overall, the study demonstrated the potential of SAW biosensors for the efficient detection of HER-2/neu, offering a promising avenue for early breast cancer diagnosis.

Breast cancer causes metabolic alteration, and volatile metabolites in the breath of patients may be used to diagnose breast cancer [107]. As a proof of concept, Yang et al. [107] developed a new breath test for breast cancer by analyzing the volatile metabolites in exhaled breath (Figure 8B). They collected air samples from breast cancer patients and non-cancer controls and used an electronic nose made of 32 carbon nanotube sensors to analyze the volatile metabolites. Machine learning techniques were employed to create predictive models for breast cancer. Using a RF algorithm, they achieved a 91% accuracy in predicting breast cancer in the test set.

One innovative way to detect breast cancer is through the use of a wearable system designed for detecting breast tumors. For instance, Elsheakh et al. [108] presented a breast cancer detection and monitoring system that utilizes microwave textile-based antenna sensors. The system consists of a wearable device that integrates the microwave antenna sensors and a portable measurement unit that wirelessly communicates with the device to collect and analyze the sensor data, as seen in Figure 8C. The proposed system aims to provide a low-cost, non-invasive, and reliable solution for the early detection and monitoring of breast cancer. The proposed system was tested on a dataset of 110 breast tissue samples, and it achieved an accuracy of 100% for breast cancer detection and classification.

As we discussed earlier, the most commonly used parameter for identifying and quantifying cells is impedance. Joshi et al. [109] demonstrated the effectiveness of single-cell impedance spectroscopy in distinguishing different types of breast cancer cells when used in conjunction with a machine learning classifier (Figure 8D). To evaluate the effectiveness of the method, the researchers pumped two types of cells through a microfluidic channel while constantly measuring the channel’s impedance throughout the entire test. The impedance measurements were then fed into a quadratic discriminant analysis (QDA) classifier, which was able to distinguish between the two types of cells with an accuracy of greater than 95% using single-feature classification. As another example, Bondancia et al. [110] developed an immunosensor to detect the cancer biomarker p53 in MCF7 breast cancer cells using electrical impedance spectroscopy. In this sensor, interdigitated electrodes were printed on bacterial nanocellulose substrates using a screen-printing technique. These electrodes were then coated with a layer-by-layer matrix of chitosan and chondroitin sulfate. On top of this matrix, a layer of anti-p53 antibodies was applied by adsorption. They also applied the DT algorithm and achieved 90% accuracy.

To enhance the detection of cancer cells, it is more logical to integrate the electrical and optical methods together. For example, Liang et al. [111] introduced a novel imaging and impedance-based single-cell analysis system called IM2Cell that enables multi-stress level mechanical phenotyping. The system is capable of simultaneously measuring both the mechanical and electrical properties of cells, providing high-dimensional information on cell structures and functions. The authors validated the imaging and impedance-based analyses separately and then combined the techniques to obtain high accuracy in predicting the characteristics of fixed and living MDA-MB-231 breast cancer cells. The authors also demonstrated IM2Cell’s ability to classify a mixture of unlabeled MCF-10A, MCF-7, and MDA-MB-231 cell lines with high accuracy. Next, IM2Cell demonstrates a 91.2% classification accuracy in a mixture of unlabeled MCF-10A, MCF-7, and MDA-MB-231 cell lines.

#### 3.2.2. Lung Cancer

In this section, we will explain two examples of different electrical sensors to detect lung cancer cells. The first example is Zhang et al. [112] developing a new biosensing strategy called SHARK (Synthetic Enzyme Shift RNA Signal Amplifier Related Cas13a Knockdown Reaction) for lung cancer detection. SHARK has broad compatibility and can be used as a portable SARS-CoV-2 biosensor with high sensitivity and selectivity, consistent with qRT-PCR results. They combined the output from the biosensors with SVM machine-learning algorithms to predict target miRNAs for (non-small cell lung cancer) NSCLC diagnosis with an accuracy of 82.81%. As another example, Van de Goor et al. [113] utilized five e-nose devices to collect breath samples from lung cancer patients and healthy controls. A total of 60 lung cancer patients and 107 healthy individuals exhaled through the e-nose for five minutes, with the participants assigned to either a training or a blinded control group. The results showed that the e-nose had a diagnostic accuracy of 83%, with a sensitivity of 83%, for discriminating between lung cancer patients and healthy controls. This study provides evidence for the feasibility and effectiveness of using a portable e-nose for accurately detecting lung cancer.

#### 3.2.3. Liver Cancer

**Figure 9:**
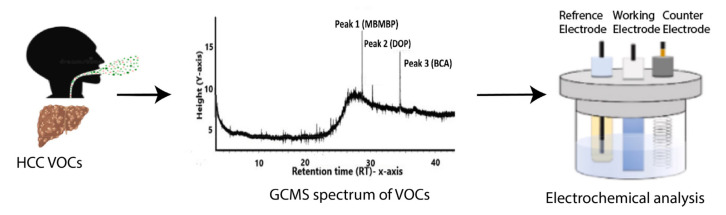
Overview of e-nose biosensor for liver cancer detection from VOCs in breath. Reprinted with permission from [114]. Copyright 2023 Elsevier.

Volatile organic compounds (VOCs) in breath are increasingly being recognized as favorable biomarkers, particularly for cancers, due to their ease of sample retrieval and specific association with early metabolic changes [114]. In the article by Nazir and Abbas [114], the use of an e-nose biosensor to detect phenol 2,2-methylene bis, 6 [1,1-D] in breath samples of hepatocellular carcinoma (HCC), which is a type of primary liver cancer, is described. Figure 9 represents an overview of the proposed model.

They conducted a screening of breath samples from patients with HCC to identify volatile organic compounds (VOCs) using gas chromatography-mass spectrometry (GC-MS). They applied unsupervised machine learning models to validate their findings. The accuracy of the developed sensor was found to be 86%, demonstrating the promising potential of this approach.

#### 3.2.4. Pancreatic Cancer

Multifrequency single-cell impedance cytometry provides multiparametric biophysical information. To demonstrate, Salahi et al. [115] developed a label-free approach to distinguish pancreatic cancer cells from their associated fibroblasts based on their biophysical properties using impedance cytometry data and machine learning algorithms. The authors demonstrate that gemcitabine treatment changes the biophysical properties of cancer cells and fibroblasts in different ways, resulting in distinguishable patterns in the impedance measurements. The approach has potential applications in cancer diagnosis, treatment monitoring, and drug development.

Combining various types of machine learning techniques has the potential to improve the accuracy of classification. By integrating different approaches, we can leverage the strengths of each method and mitigate their individual limitations, resulting in more precise and reliable classification outcomes. For instance, Honrado et al. [116] improved the classification of cancerous pancreatic cells by combining unsupervised clustering with KNN classification to detect the state of cell death experienced by the cancerous cell. The researchers collected impedance data from flow cytometry and fed it into an unsupervised clustering algorithm that operated at a hyper-dimensional level to autonomously cluster the data. The resulting metrics were then used to quantify the drug-sensitive phenotypes of cancer cells across their progression from viable to early apoptotic, late apoptotic, and necrotic subpopulations. To validate their findings, the team compared the results to those obtained through staining and found that their model was 98.4% accurate in detecting the correct phase of apoptosis in pancreatic cancer cells.

#### 3.2.5. Hematological Cancer

Recently, impedance spectrometers have been shown to generate all-inclusive lab-on-a-chip platforms to detect nucleus abnormalities. The paper, presented by Ferguson et al. [117], is a proof-of-concept study on the classification of cancerous cells using a biosensor that employs impedance-based spectroscopy to identify the type of cells based on the size of their nucleus. The biosensor consists of a microfluidic channel attached to a quartz substrate containing an ultra-wideband waveguide. The cells passing through the PDMS channel are electrically trapped using a dielectrophoretic signal, and electrical signals are collected using microwave spectroscopy. The authors used statistical elimination and feature selection techniques along with SVMs and RF algorithms to achieve a 96% accuracy on multi-class classification. The study demonstrates the potential of using machine learning in combination with microwave impedance spectroscopy for single-cell classification based on the population nucleus size, which could have significant implications for cancer diagnosis and treatment.

#### 3.2.6. Head and Neck Cancer

Currently, there is a lack of well-established effective biomarkers and convenient detection methods for predicting radioresistance. In the study by Wu et al. [118], surface-enhanced Raman spectroscopy combined with proteomics was employed to initially profile the distinct spectral patterns of the exosomes released by self-established nasopharyngeal cancer cells (NPC). They identified specific variations in protein expression during the formation of radioresistance, including collagen alpha-2 (COL1A2), which is negatively associated with DNA repair. The researchers used bioinformatics analysis and a deep learning model to accurately identify the exosomes from the radioresistance group, achieving an accuracy of 92.4%. Overall, the study provides a promising approach for identifying radioresistance-associated biomarkers in NPC.

Diagnosing cancer and other diseases using data from non-specific sensors, like electronic tongues (e-tongues), poses challenges due to the lack of selectivity and the variability of biological samples [119]. Braz et al. [119] presented an e-tongue biosensor based on microfluidic impedance flow cytometry for mouth cancer detection. Saliva samples from 27 individuals were analyzed using multidimensional projection techniques and machine learning algorithms, including the SVM with a radial basis function kernel and RF algorithms. The authors achieved an accuracy of over 80% for the binary classification of cancer vs. healthy individuals. The study suggests that the impedance data obtained with the e-tongue in saliva samples can be used for cancer diagnosis in the mouth, and the approach presented here is promising for computer-assisted diagnoses. The accuracy tended to increase when clinical information, such as alcohol consumption, was used in conjunction with the e-tongue data.

#### 3.2.7. Gynecological Cancer

A microfluidic chip for single-cell cultures utilizes self-assembled graphene oxide quantum dots (GOQDs) to facilitate high-activity single-cell cultures. This chip enables the maintenance of normal biomarker secretion in single cells and allows for efficient single cell separation at high throughputs. Consequently, it provides an ample amount of statistical data necessary for machine learning applications [120]. As a proof of concept, Wang et al. [120] developed a novel method for profiling single cells in real time using microfluidic chip technology and machine learning algorithms. They used this method to classify tumor cells based on the secreted biomarkers they produce. The microfluidic chip is designed to allow for the high-throughput analysis of single cells, enabling the measurement of multiple secreted biomarkers in real time. Then, machine learning algorithms were employed to analyze the data and classify the cells based on their biomarker profiles. The K-means strategy with machine learning was combined to analyze thousands of single tumor cell secretion data, resulting in the ability to classify tumor cells with a recognition accuracy of 95.0%.

As another example, Feng et al. [121] proposed the use of neural network-enhanced impedance flow cytometry (IFC) for the real-time, label-free, and non-invasive characterization of single cells based on intrinsic biophysical metrics. The method can obtain three intrinsic parameters (radius, cytoplasm conductivity, and specific membrane capacitance) online and in real time, achieving a significant improvement in the calculation speed. The experiments involved four cancer cell types and demonstrated a 91.5% classification accuracy. The paper suggests that this method could provide a new platform for high-throughput, real-time, and online cell intrinsic electrical characterization. Table 2 summarizes the electrical-based biosensors in conjunction with machine learning for cancer detection.

## 4. Conclusions

The pressing need to identify cancer in its earliest stages while avoiding invasive treatments has spurred the integration of innovative sensory techniques with cutting-edge machine learning algorithms. This fusion holds the potential to create a future where individuals can conveniently and promptly detect cancer within the confines of their homes. With advancements in detection technology and machine learning algorithms, our aim is to detect cancer at its early stage. Several studies have achieved highly accurate results in excess of 90% with optical biosensors, regardless of the type of cancer cell being detected or the ML algorithm used in the study. Among all the research papers analyzed for this study, most teams utilized ANNs for the machine learning aspect of their optical detection setups. On the other hand, some studies using electrical biosensors achieved slightly lower, yet consistently high, results when compared to the teams that employed optical biosensors. Slightly more than half of these teams recorded an accuracy greater than 90%, while the remaining teams had accuracies that were slightly lower. Most of these teams used SVMs to incorporate machine learning into their research, with ANNs being used to a lesser extent than in the optical detection teams. With further progress and advancements in these methodologies, we can hope for continuous improvements in the results and eventually strive towards a cancer-free future.

Biosensors are analytical devices that combine biological components, known as bioreceptors, with transducers to detect specific biological or chemical analytes. Despite the significant advancements, biosensors still face challenges related to the bioreceptor immobilization matrices, immobilization efficiency, and predicting responses in complex matrices. Machine learning (ML) can play a vital role in addressing these issues. For instance, ML models can assist in selecting the most suitable immobilization matrix for a specific bioreceptor by considering factors such as the bioreceptor type, analyte characteristics, and environmental conditions. This predictive capability helps researchers optimize the immobilization process and anticipate and correct deviations in sensor responses. Additionally, ML can aid in sensor calibration and data fusion, enhancing the accuracy and reliability of biosensor readings by continuously monitoring and adjusting the sensor responses based on historical data and real-time measurements.

In summary, biosensors are essential analytical tools with some inherent limitations. ML can offer valuable solutions by assisting in the selection of immobilization matrices for bioreceptors and improving sensor calibration and data fusion processes. These ML-driven interventions enhance the overall performance and reliability of biosensors, making them more effective in applications such as cancer cell detection and other complex analytical tasks.

## Figures and Tables

**Table 1:** Comparison of different optical-based biosensors with ML analysis for cancer cell detection.

Authors	Cancer Cell Type	Biosensor Type	ML Algorithm	Results (%)
Kumar et al. [64]	Breast Cancer	(Surface plasmon resonance) SPR sensor	ANN	MSE = 0.01525 percentage error of 2%
Verma et al. [65]	Breast Cancer	SPR sensor	ANN	MSE = 0.116
Jin et al. [67]	Breast Cancer	Fluorescence sensor	ANN	ACC = 100
Pala et al. [68]	Breast Cancer	CMOS imaging sensor	ANN	ACC = 99.65
Hashemzadeh et al. [70]	Lung Cancer	Olympus fluorescence microscope	ANN	ACC = 98.37
Sui et al. [71]	Lung Cancer	Fluorescence sensor	CNN	ACC = 91–95
Nguyen et al. [72]	Lung Cancer	Gap plasmonic color sensors	Convolutional neural network (CNN)	ACC = 89
Wei et al. [73]	Lung Cancer	Two-dimensional (2D) light-scattering	SVM	ACC = 99.87
Ahmad et al. [75]	hTERT-immortalized human mammary epithelial cells (IMEC WT) Xenograft-derived primary tumor cells (XD) Lung metastasis-derived cells (MD)	Fluorescence microscopy Image-based sensor	CNN	ACC = 99.4
Lin et al. [76]	Lung and Colon Cancer	Localized plasmonic sensor	SVM	ACC = 85.72
Park et al. [77]	Lung Cancer	Surface-enhanced Raman spectroscopy (SERS)	Principal component analysis (PCA)	Sensitivity = 95.3
Ko et al. [78]	Pancreatic Cancer	Image-based multichannel nanofluidic system	LDA	AUC = 0.81
Li et al. [79]	Colorectal Cancer	Image-based 3D porous microfluidic chip	RF	ACC = 91.4
Cheng et al. [80]	Liver Cancer	SERS sensor	ANN	ACC = 91
D’Orazio et al. [81]	Colorectal Cancer	image-based time-lapse microscopy	ANN	ACC = 86.77
Saren et al. [82]	Gastrointestinal Cancer	Quantum dot (QD)-labeled biofilms	Principal component analysis (PCA)	ACC = 94
Mencattini et al. [85]	PKD, which may cause Liver, Colon, and Kidney Cancer	Image-based time-lapse microscopy	ANN	ACC = 88
Liu et al. [87]	Cervical Cancer	Image-based high-content VFC (video flow cytometry)	CNN + SVM	ACC = 90.8
Kim et al. [88]	Ovarian Cancer	Nanosensor array	SVM	ACC = 95
Pirone et al. [89]	Endometrial cancer	Holographic flow cytometry (DHFC)	LDA	ACC = 96
Rodrigues et al. [91]	Prostate Cancer	Genosensors	SVM and LDA	ACC = 99.9
Linh et al. [92]	Prostate and Pancreatic Cancers	SERS sensor	ANN	ACC = 99.4
McRae et al. [93]	Prostate and Ovarian Cancer	Bio-nanochip sensor	ANN	AUC = 0.94
Hasan et al. [94]	Brain Cancer	Image-based time-lapse images	SVM + RF + NBC	ACC > 82
Hossain et al. [97]	Brain Cancer	Sensor-based microwave brain imaging (SMBI)	CNN	ACC = ~90
Koowattanasuchat et al. [99]	Leukemia Cancer	Colorimetric biosensors	RF + SVM	ACC = 90
Uslu et al. [102]	Lymphoma and Leukemia Cancer	Microscope images	RF	ACC = 87.4
Sarkar et al. [103]	Hematological Cancer	Droplet microfluidics-based cytotoxicity imaging approach	ANN	ACC = 94
Li et al. [104]	Epithelial Cancer	Image-based microfluidic channel	CNN	ACC > 95

**Table 2:** Comparison of different electrical-based biosensors with ML analysis for cancer cell detection.

Authors	Cancer Cell Type	Biosensor Type	ML Algorithm	Result (%)
Ahuja et al. [105]	T47D cancer cells (Type of Breast cancer)	Microfluidic device impedance cytometry	SVM	ACC = 95.9
Sountharrajan et al. [106]	Breast Cancer	Surface acoustic wave (SAW) biosensor	SVM	ACC = 79.25
Yang et al. [107]	Breast Cancer	Nanotube sensors	Random forest (RF)	ACC = 91
Elsheakh et al. [108]	Breast Cancer	Microwave textile-based antenna sensors	CatBoost (Type of gradient boosting)	ACC = 100
Joshi et al. [109]	Breast Cancer	Microfluidic channel sensor	Quadratic discriminant analysis (QDA)	ACC > 95.3
Bondancia et al. [110]	Breast Cancer	Immunosensor	DT	ACC = 90
Liang et al. [111]	Breast Cancer Combination of electrical and optical-based sensors	Impedance-based sensor	(Linear discriminant analysis) LDA + SVM	ACC = 91.2
Zhang et al. [112]	Lung Cancer	SHARK (Synthetic Enzyme Shift RNA Signal Amplifier Related Cas13a Knockdown Reaction)	SVM	ACC = 82.81
Van de Goor et al. [113]	Lung Cancer	E-nose biosensor	ANN	ACC = 93
Nazir and Abbas [114]	Liver Cancer	E-nose biosensor	Unsupervised ML	ACC = 86
Salahi et al. [115]	Pancreatic Cancer	Microfluidic device impedance cytometry	SVM	ACC = 93.7
Honrado et al. [116]	Pancreatic Cancer	Microfluidic device impedance cytometry	KNN	ACC = 98.4
Ferguson et al. [117]	Jurkat Cells (Type of Leukemia Cancer)	Microfluidic device	RF + SVM	ACC = 96
Wu et al. [118]	Nasopharyngeal Cancer	Surface-enhanced Raman spectroscopy	ANN	ACC = 92.4
Braz et al. [119]	Oral Cancer	E-tongue biosensor	RF + SVM	ACC = 80
Wang et al. [120]	Ovarian, Kidney, Breast, Lymph Cancer	Microfluidic chip	K-means	ACC = 95
Feng et al. [121]	Breast, Cervical, Lung, Leukemia Cancer	Impedance flow cytometry (IFC)	ANN	ACC = 91.5

## Data Availability

The data presented in this study are available on request from the corresponding author. The data are not publicly available due to ethical constraints.
